# The Eukaryotic Elongation Factor 1 Alpha (eEF1α) from the Parasite *Leishmania infantum* Is Modified with the Immunomodulatory Substituent Phosphorylcholine (PC)

**DOI:** 10.3390/molecules22122094

**Published:** 2017-11-29

**Authors:** Thomas Timm, Giada Annoscia, Jochen Klein, Günter Lochnit

**Affiliations:** 1 Protein Analytics, Institute of Biochemistry, Faculty of Medicine, Justus-Liebig-University Giessen, Friedrichstrasse 24, 35392 Giessen, Germany; thomas.timm@biochemie.med.uni-giessen.de; 2 Department of Veterinary Medicine, University of Bari, Str. prov. per Casamassima km 3, Valenzano, 70010 Bari, Italy; annoscia.giada@gmail.com; 3 Department of Pharmacology, Goethe University College of Pharmacy, 60438 Frankfurt, Germany; klein@em.uni-frankfurt.de

**Keywords:** *Leishmania infantum*, promastigote, proteomics, phosphorylcholine, immunomodulation, immunofluorescence

## Abstract

Proteins and glycolipids have been found to be decorated with phosphorylcholine (PC) both in protozoa and nematodes that parasitize humans and animals. PC epitopes can provoke various effects on immune cells leading to an immunomodulation of the host’s immune system that allows long-term persistence of the parasites. So far, only a limited number of PC-modified proteins, mainly from nematodes, have been identified. Infections caused by *Leishmania* spp. (e.g., *L. infantum* in southern Europe) affect about 12 million people worldwide and are characterized by a wide spectrum of clinical forms in humans, ranging from cutaneous to fatal visceral leishmaniasis. To establish and maintain the infection, these protozoa are dependent on the secretion of effector molecules into the host for modulating their immune system. In this project, we analyzed the PC modification of *L. infantum* promastigotes by 2D-gel based proteomics. Western blot analysis with the PC-specific antibody TEPC-15 revealed one PC-substituted protein in this organism, identified as eEF1α. We could demonstrate that the binding of eEF1α to one of its downstream effectors is dependent on its PC-modification. In this study we provide evidence that in this parasite the modification of eEF1α with PC may be essential for its function as an important virulence factor.

## 1. Introduction

Leishmaniasis, a vector-borne worldwide-distributed parasitic disease, is caused by dimorphic protozoan flagellates of the genus *Leishmania*. This organism alternates between two different stages: the promastigote lives extracellular in its vector, the phlebotomine sandfly (Diptera, Psychodidae), and the amastigote resides within the phagolysosome of mononuclear macrophages of mammalian hosts. The disease is characterized by diversity and complexity, presenting a wide spectrum of clinical forms in humans, ranging from cutaneous leishmaniasis (CL) to fatal visceral leishmaniasis (VL) [1,2].

Recent studies have reported the diffusion of new “hot spots” of canine leishmaniosis in previously non-endemic areas of the northern United States and some provinces of southern Canada and northern Europe [3,4,5]. Leishmaniosis seems to spread because of a combination of factors: environmental/climatic changes, factors related to the immune status of the host and drug resistance [6,7]. Therefore, over the last decades, considerable efforts have been directed towards the research into new proteinacious targets for the development of an effective therapy against human VL.

**Figure 1:**
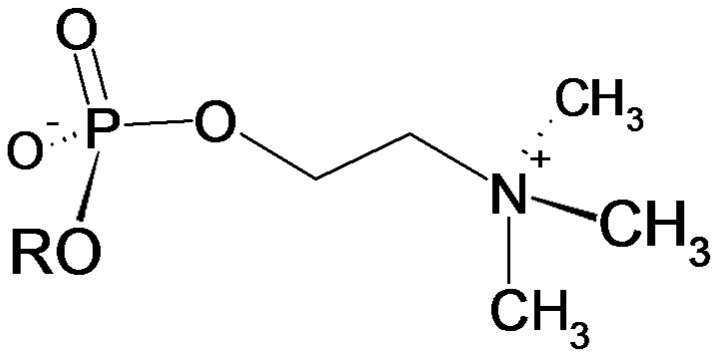
Structure of the phosphocholine functionality with R being an *N*-glycan or the sidechaine of an amino acid.

Phosphorylcholine (PC, Figure 1) has been recognized as a common antigenic determinant in many important disease-causing parasites, such as, gastrointestinal and filarial nematodes [8] but also in protozoa like *Trypanosoma* and *Leishmania* [9].

PC-bearing antigens have been found to possess immunomodulatory capacity and to interfere with key proliferative signaling pathways in B- and T-cells, dendritic cell maturation and mast cell degranulation, thus facilitating the survival of parasites in their hosts [8,10,11,12,13,14,15,16,17]. Therefore, these effects could contribute to the observed modulated cytokine levels and impairment of lymphocyte proliferation. Detailed data on the different types of PC-carrying biomolecules as well as their biosynthesis, however, are limited and have only been reported in the last few years in nematodes [8,18,19,20,21].

Structural analyses of nematode-derived molecules with PC epitopes focused, so far, on glycolipids and glycoprotein glycans. It could be shown that glycosphingolipids of the pig parasitic nematode, *Ascaris suum*, are characterized by the presence of a phosphodiester-bound PC-substituent which has been assigned to C-6 of the central *N*-acetylglucosamine (GlcNAc) residue of an arthro-series carbohydrate core [22]. Furthermore, some glycolipid species were found to carry phosphorylethanolamine linked to C-6 of an adjacent mannose residue in addition to PC [23,24]. Comparable glycosphingolipids have been verified in different nematodes, including *Nippostrongylus brasiliensis* [25], *Litomosoides sigmodontis* [26,27], *Onchocerca volvulus* and *Setaria digitata* [27], indicating that arthro-series glycosphingolipids carrying, in part, PC substituents represent highly conserved glycolipid markers within the nematode phylum. A biosynthetic route homologous to *A. suum* glycosphingolipids was also confirmed for the free-living nematode *Caenorhabditis elegans* [23,28,29].

Analogous analyses of the PC-substituted glycoprotein ES-62, an excretory/secretory (ES) product of *Acanthocheilonema viteae*, indicated that the zwitterionic substituent is linked via *N*-glycans to the polypeptide backbone [30,31]. Mass spectrometric analysis of the respective *N*-linked glycans revealed the presence of trimannosyl *N*-glycan variants, carrying between one and four terminal GlcNAc residues [32]. Only this type of glycans was found to be substituted with PC-moieties which could be again assigned to C-6 of terminal GlcNAc residues [33,34]. Comparative studies of *N*-glycans present in extracts of *A. viteae*, *Onchocerca gibsoni* and *O. volvulus* confirmed a high conservation of such PC-substituted *N*-glycans within filarial parasites [35]. For *C. elegans*, two types of PC-substituted *N*-glycans have been reported so far: (1) a pentamannosyl-core structure carrying up to three PC-residues [36] and (2) trimannosyl-core species elongated by GlcNAc residues substituted at C-6 with PC [33]. Furthermore, combinations of both types of structural motifs [19] as well as the occurrence of extended glycan structures with composition of *PCho*_1-2_dHex_0-2_Hex_3-5_HexNAc_3-7_ have been reported [18]. For *A. suum* hybrid-type bi- and triantennary *N*-glycans substituted with PC have been described [21].

PC-modified proteins were also detected in different developmental stages of the malarial blood parasite *Plasmodium falciparum* [37]. Fourteen putative proteins carrying the PC modification were identified, among them, proteins that are located on the surface of the parasite, or are involved in metabolism. Erythrocyte membrane protein 1 (EMP1, a member of the *var* gene family), together with the heat shock protein 70 (HSP-70), was detected in every *P. falciparum* stage of the erythrocyte pathway. In *P. falciparum*, *var* genes encode adhesive proteins that are transported to the surface of infected erythrocytes, thereby acting as major virulence determinants for immune evasion [38,39]. There is increasing evidence that HSP-70 could play an important role in the life cycle of *P. falciparum* both as a chaperone and as immunogen [40]. In the merezoite-stage, only one surface protein (EMP1, P154varH), was detected.

The eukaryotic elongation factor-1α (eEF1α) is an enzyme that catalyzes the GTP-dependent binding of aminoacyl-tRNA to the A-site of ribosomes during protein synthesis and is involved in the capture of deacylated tRNA [41,42]. Furthermore, eEF1α was found to serve as a central hub in protein networks with hundreds of interacting partners [43,44]. *Leishmania* eEF1α was found to bind and activate the Src-homology 2 domain containing protein tyrosine phosphatase-1 (SHP-1), a protein known to be involved in the macrophage inactivation pathogenesis of leishmaniasis [41]. Additionally, eEF1α was found in *Leishmania* exosomes and identified as an important factor for immunosuppression and priming host cells for *Leishmania* invasion [41,45].

In this study, we identified eEF1α as the only PC-positive protein found in *Leishmania infantum* MON-1 by a 2D-gel proteomic approach. Furthermore, we confirmed the presence of PC modifications by quantitative determination of its choline content. Additionally, we localized the PC epitopes within procyclic and stationary phase promastigotes by confocal microscopy. Finally, we were able to demonstrate that the interaction of *L. infantum* EF1α and human SHP-1 is dependent on the PC modification of EF1α.

## 2. Materials and Methods

### 2.1. Cultivation of Leishmania infantum Promastigotes

*L. infantum* zymodeme MON-1 promastigotes were cultured in Tobie–Evans modified medium at 24 °C [46]. Promastigotes at day 3 or 10 of culture were used. The liquid phases were centrifuged at 1000× *g* for 5 min at 4 °C. Supernatant was then discarded and the pellet was resuspended in phosphate buffered saline (PBS) pH 7.2, and washed 3 times by centrifugation at 1000× *g* for 10 min.

### 2.2. Immunofluorescence

*L. infantum* promastigotes were washed twice in PBS before fixation in 200 µL of 1% formaldehyde in PBS for 30 min at room temperature (RT). After a PBS wash, the cells were permeabilized by resuspension in 200 µL of 0.1% Triton X-100 in PBS for 10 min. Following an additional PBS wash, the cells were resuspended in 200 µL of 0.1 M glycine in PBS and incubated for a further 10 min at RT before being washed in PBS. Glass slides were washed with 70% ethanol and coated with a 0.01% solution of poly-l-lysine (0.1% stock; Sigma Alrich, Taufkirchen, Germany), and the fixed, permeabilized cells were then left to sediment and adhere to the surfaces of these polylysine-coated slides for 15 min at RT.

Monoclonal mouse TEPC-15 (Sigma Aldrich, Taufkirchen, Germany) diluted 1:1000 in TB buffer (0.1% (*v*/*v*) Triton X-100, 0.1% (*w*/*v*) bovine serum albumin (BSA) in PBS), was added to the slide and incubated with the cells overnight at 4 °C. After a 10-mL PBS wash, cells were incubated in the dark for 1 h at RT with FITC-conjugated secondary antibodies (LifeTechnologies, Darmstadt, Germany) diluted 1:500 in TB buffer. Unbound secondary antibody was washed away with 1.5 mL PBS (3-times 0.5 mL).

The cells were then covered with 10 µL of Vectashield mounting medium with DAPI for staining of the cellular DNA (Vector Laboratories, Burlingame, CA, USA).

Preparations were examined with a Zeiss LSM 710 confocal microscope using a 488 nm laser for FITC and a 405 nm laser for DAPI. The instrument control and image analysis were done with the software ZEN (version 2012, blue edition; Zeiss, Wetzlar, Germany).

### 2.3. Protein Isolation

Parasites were prepared as described in section “Cultivation of *Leishmania infantum* promastigotes”. Approximately 10 mg of pelleted promastigotes were homogenized in 30 μL 0.2% (*w*/*v*) sodium dodecylsulfate (SDS; Roth, Karlsruhe, Germany) and boiled for 5 min at 95 °C. After cooling on ice, the sample was extracted with 370 µL lysis buffer consisting of 6 M urea (Sigma, Taufkirchen, Germany), 2 M thiourea (Sigma, Taufkirchen, Germany), 1% Triton X-100 (Fluka, Selze, Germany), 65 mM dithiothreitol (DTT; Fluka, Selze, Germany), 0.5% IPG-buffer pH 3–10 (GE Healthcare, Freiburg, Germany), 0.1 mM phenylmethylsulfonylfluoride (PMSF; Sigma, Taufkirchen, Germany), and Protease Inhibitor Cocktail for general use (Sigma Alrich, Taufkirchen, Germany). Sample homogenization was done by four pulses of 30 s in a bullet blender using 1.4 mm stainless steel beads (Next Advance, New York, NY, USA) with intermediate cooling on ice and centrifugation (20,000× *g*, 1 h at 4 °C). To remove lipid contaminants and SDS proteins were precipitated with chloroform/methanol (1:4 by *v*/*v*). For isoelectric focusing, the protein pellet was dissolved in 100 μL lysis buffer containing 4% 3-3′-(Cholamidopropyl)-3,3-dimethylammoniumpropylsulfat (CHAPS; Roth, Karlsruhe, Germany) instead of Triton X-100 and 2% IPG-buffer pH 3–10 [47].

### 2.4. Two-Dimensional Gel Electrophoresis and Detection of PC-Modified Proteins

Two-dimensional separation, Western blotting of proteins and detection of PC-modified proteins was performed as described in [47]. For preparative gels 0.4–0.5 mg, and for Western blot analysis 25 µg of the extracted protein were loaded.

eEF1α was detected by the specific antibody PA5-17213 (Thermo Scientific, Dreieich, Germany, 1:2000 dilution) with horseradish peroxidase conjugated anti-rabbit Ig (DakoCytomation, Glostrup, Denmark, 1:2000) as secondary antibody. Proteins recognized by the antibodies were visualized by enhanced chemiluminescence using the ECL SuperSignal kit (GE Healthcare, Solingen, Germany). The corresponding protein spots were excised from preparative gels with the ExQuest™ Spot Cutter (Bio-Rad, Munich, Germany) and transferred into 96-well plates (Greiner Bio-One, Frickenhausen, Germany).

### 2.5. Tryptic in-Gel Digestion of Proteins, Matrix-Assisted Laser-Desorption Ionization Time-of-Flight Mass Spectrometry (MALDI-TOFMS) and Database Search

Performed as described in [47].

### 2.6. Choline Quantification

For the quantitation of choline-substitution of eEF1α, 400 µg of total protein from *L. infantum* were separated on a 2D-gel as described above and stained with Flamingo^TM^ according to the instructions of the manufacturer (BioRad, München, Germany). The absolute amount of eEF1α was determined by densitometric analysis using Quantity One 4.6.2 (Bio-Rad, Munich, Germany) using lysozyme (14.3 kDa, 5 µg), β-lactoglobulin (18.4 kDa, 3 µg), aldolase (36 kDa, 2 µg), and bovine serum albumin (66 kDa, 1 µg; all from Sigma Aldrich, Taufkirchen, Germany) as standards (see results). The spots containing 1.9 and 2.1 respectively µg of EF1α were cut from the gels and tryptic digestion was performed as described above. PC residues were removed from the peptides by cleavage of the phosphodiester bonds with hydrogen fluoride: one sixth (6.26/7.25 pmol) of the tryptic digest of eEF1α was lyophilized, dissolved in 50 µL of hydrofluoric acid (48%, Merck, Darmstadt, Germany) and incubated on ice overnight. The sample was dried under a stream of nitrogen, dissolved in 500 µL of water and lyophilized. Choline was measured by HPLC according to published methods [48].

### 2.7. Co-Purification of Leishmania EF1α with Human SHP-1

Approximately 50 mg of pelleted promastigotes were extracted with 1 ml RIPA buffer (25 mM TRIS-HCl pH 7.6, 150 mM NaCl, 1% NP40, 1% sodium deoxycholate, 0.1% SDS), half of the extract was supplemented either with 2 µg of GST-SHP-1 (Jena Bioscience, Jena, Germany) alone or in presence of phosphorylcholine (5 mM final concentration; Sigma Aldrich, Taufkirchen, Germany). To get rid of residual glutathione from the preparation of GST-SHP-1, the buffer was exchanged three times by ultrafiltration (10 kDa cutoff; Amicon, Darmstadt, Germany). After an incubation on ice for three hours, the solutions were separately passed two times through 50 µL glutathione agarose (Jena Bioscience, Jena, Germany), pre-incubated with RIPA or RIPA/PC respectively. Proteins were then eluted with 80 µL of twofold concentrated Laemmli sample buffer and subjected to SDS-PAGE. Western blotting and detection was performed as described above using 3% BSA in TBS-T instead of Roti-Block.

### 2.8. Staining of Proteins on Western Blot Membranes

To visualize all the proteins transferred to the PVDF membranes these were incubated with black Fount India Ink (Pelikan, Hannover, Germany; diluted 1:1000 in PBS-T) for at least five hours, washed five times with pure water and dried.

## 3. Results

### 3.1. 2D-Gelelectrophoresis and Western Blot Analyses

**Figure 2:**
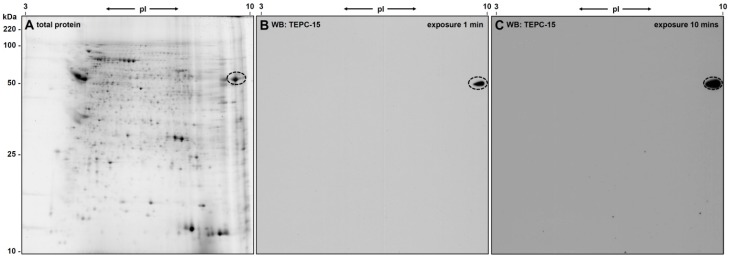
2D-Gel electrophoretic separation of *L. infantum* proteins. (**A**) Gel stained with Flamingo for total protein visualization and (**B**,**C**) ECLs of a Western blot probed with the PC-specific antibody TEPC15. One spot is detected as being PC-modified, even if the film is overexposed: image (**C**) is exposed for 10 min, although the spot is already visible after 1 min (**B**). The corresponding spot in (**A**) is labelled with a circle.

The *L. infantum* promastigote protein extract was separated by 2D-gel electrophoresis and proteins were visualized by Flamingo staining (see Figure 2A). A weak blockage in the iso-electric focusing (IEF) could be observed in the basic region of the gel probably due to nucleic acids in the sample; however, protein spots were clearly resolved even at the very basic region of the gel.

PC-epitopes were detected by western blotting using the PC-specific antibody TEPC-15. Only a single ellipsoid spot was found after ECL (see Figure 2B) even after longer exposure (ten times) of the film (Figure 2C). The ellipsoid form of the spot gave first indication for the presence of several isoforms/post-translational modifications of the protein(s) beyond the spot.

The PC-positive spot from the western blot could be clearly matched to a protein spot on the Flamingo stained gel with an apparent molecular weight of approx. 50 kDa and a pI of approximately 9.5.

### 3.2. Identification of eEF1alpha

**Figure 3:**
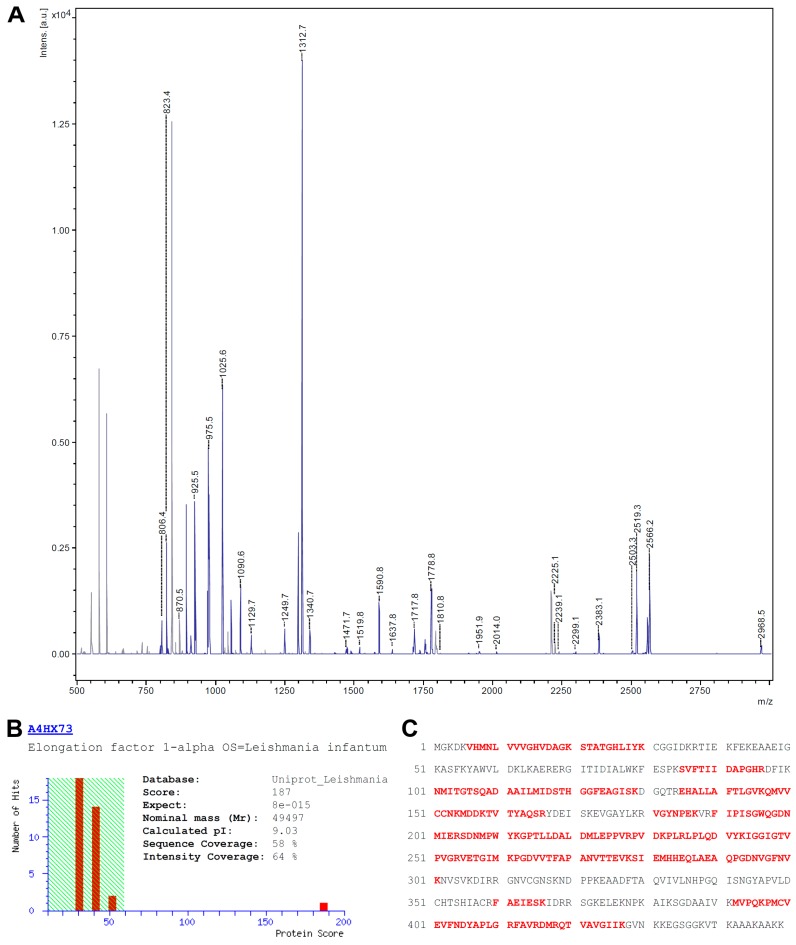
Identification of eEF1α. (**A**) MS-Spectrum of the tryptic digest of the processed protein spot. Peaks matching to the sequence of eEF1α are shown in blue; (**B**) Identification of eEF1α with a MOWSE score of 187 (scores above 59 are significant; *p* < 0.05). The theoretical values for the molecular weight and pI correspond to the observed ones; (**C**) Sequence of eEF1α with peptides identified by database search highlighted in red.

For protein identification, the complete PC-positive spot was isolated from the Flamingo-stained gel, digested with trypsin and subjected to MALDI-TOF-MS peptide fingerprint analysis. The resulting peptide mass list from the MS spectra acquired (see Figure 3A) was used for a database search using the MASCOT search engine and an in house installed Leishmaniinae database extracted from Uniprot.

The database search presented only one protein hit (accession no A4HX73) with statistical significance (*p* < 0.05) and with a MOWSE score of 187, the elongation factor 1 alpha (eEF1α; see Figure 3B). The matched peptides (highlighted in red in Figure 3C) covered 58% of the amino acid sequence with an intensity coverage of 64%. A re-search with unmatched masses from the initial search yielded no significant identification, excluding the presence of a second protein in that particular spot. A re-search including PC as an optional modification on serine, threonine, tyrosine, lysine and cysteine residues did not lead to an identification of PC-modified peptides. From the amino acid sequence of the database entry a molecular weight of 49.5 kDa and a pI of 9.03 could be calculated. The observed molecular weight (50 kDa) fits well to the theoretical value whereas the apparent pI (approx. 9.5) is a slightly more basic than expected.

### 3.3. Western Blot Analysis with Anti-eEF1α

**Figure 4:**
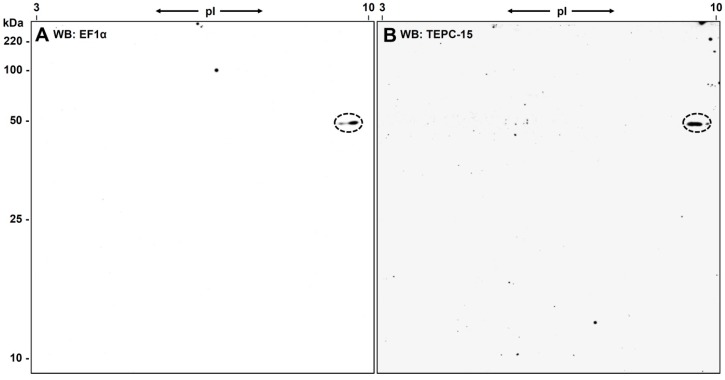
2D-Western blot analyses with eEF1α- and PC-specific antibodies. (**A**) ECL of a Western blot probed with PA5-17213, an eEF1α-specific antibody. The elongation factor is detected at its expected molecular weight (49.5 kDa) but at a slightly higher pI (9.5 compared to an expected pI of 9.03). There is also a second spot of the same size but at lower pI detectable, representing another protein species of eEF1α (**B**). Both spots of eEF1α shown in (**A**) are recognized by the PC-specific antibody TEPC-15. Note that this *Leishmania* were cultivated for ten days but display no difference in PC-substitution compared to the ones cultivated only for three days.

To confirm that the PC-positive spot in the 2D-gel is really eEF1α, a Western blot experiment was performed using an eEF1α-specific antibody (PA5-17213; Thermo Scientific, Dreieich, Germany). In this experiment we also used *L. infantum* parasites cultivated for ten days instead of three to see whether we get a different pattern of PC-substitution. ECL detection revealed one dominant spot next to a weaker signal with nearly identical molecular weight exactly corresponding to the TEPC-15-positive signal in a parallel Western blot experiment (see Figure 4A,B). The two spots in the PA5-17213 blot confirmed the assumption that more than one eEF1α proteoforms are present in the *L. infantum* promastigotes.

### 3.4. Verification of the Phosphorylcholine Substitution of eEF1α and Choline Quantitation

To verify a phosphorylcholine modification of eEF1α, we made use of a HPLC-based method developed by the group of J. Klein [47]. In this method, the retention time of choline was observed on an ion-exchange column. Selective detection of choline was performed with an enzymatic reactor containing immobilized choline oxidase. The amount of choline was measured by the formation of H_2_O_2_, which was then detected electrochemically.

For the analysis of choline, the tryptic peptides from eEF1α were treated with hydrogene fluoride (HF) which quantitatively cleaves phosphomono- and -diester bonds [49].

**Figure 5:**
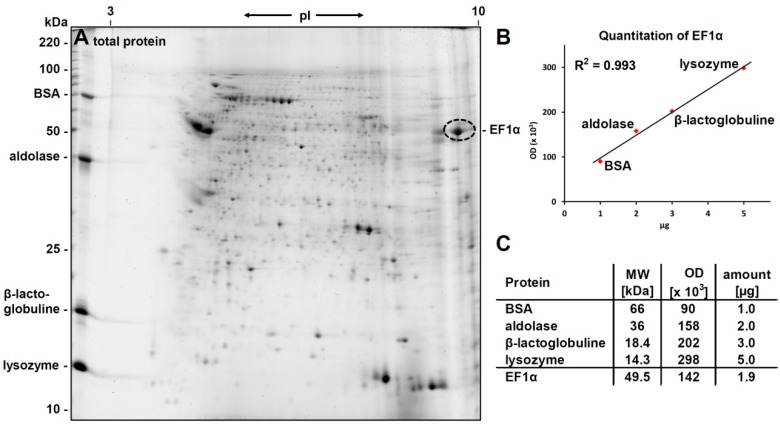
Quantitation of the extracted eEF1α. (**A**) Gel stained with Flamingo for total protein visualization. To quantify individual spots by densitometric analysis, four proteins with specific amounts were run along the sample in the second dimension; (**B**) The plot shows the linear correlation of optical densities of the four proteins with their nominal amount; (**C**) The table displays the data for the calibration proteins and the derived amount of eEF1α contained in 400 µg of total protein extract.

For quantitative analysis of the phosphorylcholine substitution, the amount of eEF1α in the 2D-gel was determined densitometrically by running different amounts of lysozyme, β-lactoglobulin, aldolase and bovine serum albumin in the second dimension parallel to the sample. After staining the 2D-gel with flamingo, a calibration curve was generated. Starting with 400 µg of total protein from *L. infantum* promastigotes, approx. 1.9 µg eEF1α were found in the PC-positive spots used for choline quantification (see Figure 5).

For choline quantitation, tryptic peptides from 310 ng (approx. 6.26 pmol) eEF1α were treated with HF and 1.31 pmol choline were obtained. A repetition of the experiment yielded similar values (2.1 µg EF1α per 400 µg total protein, and 1.32 pmol choline per 7.25 pmol of EF1α). This indicated a phosphorylcholine substitution degree of around 20% for eEF1α (19.6% ± 1.4%).

### 3.5. Immunohistological Localization of PC Epitopes in L. infantum Promastigotes by Confocal Microscopy

For immunohistological localization, *L. infantum* promastigotes were permeabilized with TritonX-100 and fixed on polylysine-coated slides. Again, the PC-specific antibody TEPC-15 was used as primary antibody and a FITC-conjugated secondary antibody was used for immunofluorescence-based localization of the PC epitopes. Additionally, the nuclei and kinetoplasts were stained with DAPI.

**Figure 6:**
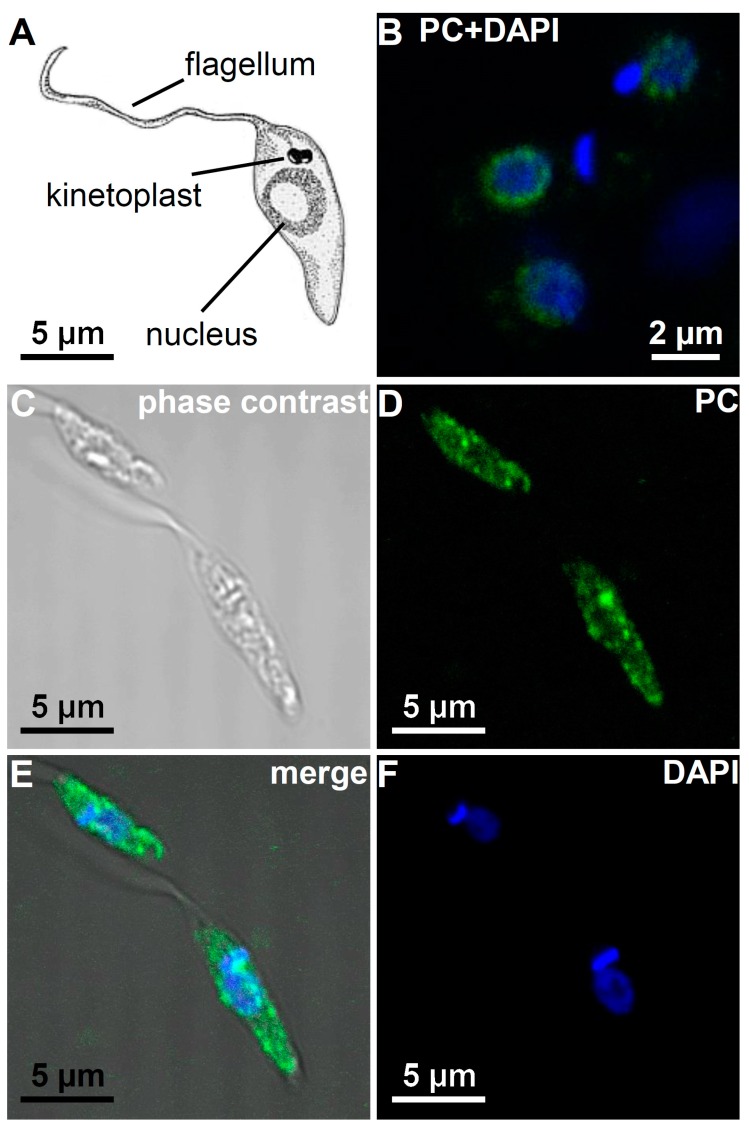
Localization of the PC epitope by confocal microscopy in *L. infantum* promastigotes. (**A**) Drawing of a *L. infantum* promastigote (modified from valleyveterinarygroup.com); (**B**) Confocal image of three *L. infantum* promastigotes after three days in culture. The nucleus and the kinetoplast are stained with DAPI (shown in blue). The PC epitope is recognized by the TEPC-15 antibody as a pearly ring around the nucleus (shown in green); (**C**–**F**) Confocal images of two *L. infantum* promastigotes after ten days in culture. The PC epitope is now localized throughout the whole cytoplasm (**D**); still having a rather dotted and non-homogeneous appearance; Image (**E**) shows a merge of the phase contrast image (**C**) with the PC- (**D**) and DAPI stain (**F**).

The PC epitopes were found to be restricted in the perinuclear region in promastigotes cultured for three days. No other organelles like the kinetoplasts or the flagella were found to carry these epitopes (see Figure 6). Interestingly, the PC epitopes showed a regular pearly distribution around the nucleus (see Figure 6B). As a control, cells were incubated only with the secondary antibody giving no signal (data not shown). However, in *Leishmania* that where cultivated for ten days the distribution of the PC epitope differed; now the PC-epitope was also found in the whole cytoplasm (Figure 6D,E). 

To exclude the possibility that with longer cultivation additional proteins became modified with PC, we extracted *L. infantum* promastigotes cultured for ten days, performed a 2D-gel separation of the proteins and confirmed in a Western blot analysis with TEPC-15 that still only eEF1α was detected as PC-modified protein (see Figure 4).

### 3.6. Molecular Function of the PC Modification of EF1α 

**Figure 7:**
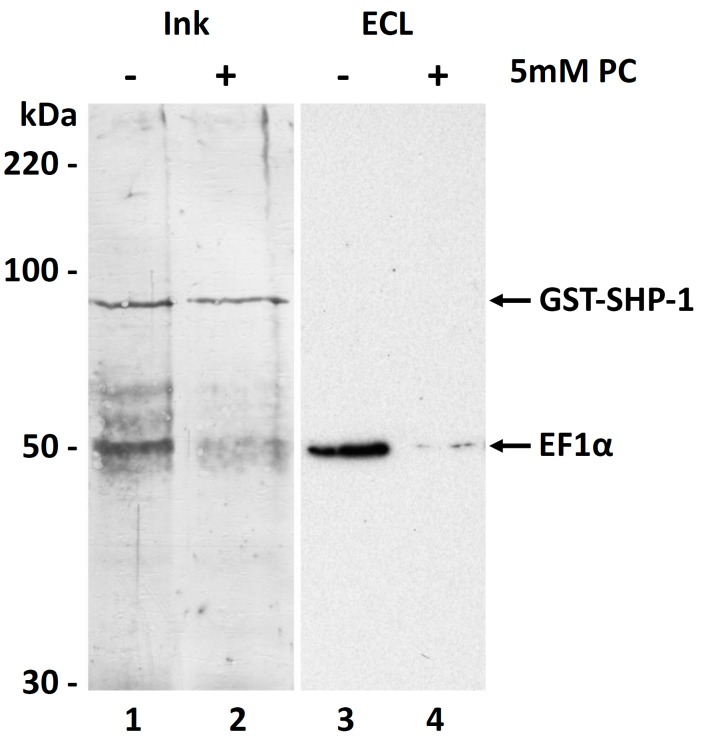
Interaction of *L. infantum* EF1α with human SHP-1. *Leishmania* EF1α co-purifies with human GST-SHP-1 as detected in a Western blot by the eEF1α-specific antibody (lane **3**). This interaction is almost completely abolished in presence of 5 mM phosphorylcholine (lane **4**). Lanes **1** and **2** show an Ink-staining of the proteins co-eluting with the GST-tagged phosphatase SHP-1 directly on the PVDF membrane. Whereas the SHP-1 (~90 kDa) is visible in both lanes at similar amounts, a band corresponding to EF1α (~50 kDa) is much stronger in lane 1 than in lane 2, were PC interrupts their interaction.

In 2012, Silverman and Reiner reported that elongation factor 1 alpha, among other proteins, was delivered into host macrophages via exosomes [45]. In previous work, the same group describes that *Leishmania* EF-1α specifically activates the Src homology-2 domain-containing phosphatase-1 (SHP-1), but recombinant protein lacks that function [41]. So we were interested in finding out if the interaction of EF-1α with SHP-1, which leads to its activation, is influenced by the PC modification of EF-1α. To address this question, we incubated GST-tagged human SHP-1 with a crude extract of *L. infantum* in presence or absence of 5 mM free phosphorylcholine. SHP-1 and its potential binding partners were then captured utilizing its tag and analyzed by Western blotting. As shown in Figure 7, EF1α is co-purifying with GST-SHP-1 as expected, but more importantly, this interaction is strongly reduced when PC is included in the extract. This supports the hypothesis that the binding of EF-1α to SHP-1 is dependent on the modification of EF1α with PC. In addition, this confirms that EF1α is really the PC-modified protein and that we were not misled by a molecule co-migrating with the elongation factor in 2D-Gel analysis.

## 4. Discussion

In this study, we detected a single PC-substituted protein in *L. infantum* promastigotes, which was definitely identified as eEF1α. eEF1α represents about 0.5% of the parasite’s total protein extract. The substitution of eEF1α with the immunomodulatory substituent PC was confirmed by choline quantification to a degree of at least 20%. By confocal microscopy, the PC epitope was found to be localized with a pearly distribution in the perinuclear region in shortly cultivated parasites, whereas after longer cultivation the PC-modified protein is found in the whole cytoplasm. By this, the parasite probably prepares the secretion of the PC-modified elongation factor as reported by Silverman and Reiner [45]. After release into the host cell, this particular modification may be involved in the regulation of the binding of EF1α to its effector SHP-1.

### 4.1. PC as An Immunomodulatory Substituent

To escape the immune response, parasites have developed various strategies: the more passive strategies comprise intracellular localization, molecular camouflage by decoration with host-derived molecules or mimicry by synthesizing epitope structures resembling host molecules. More active strategies include the release of compounds degrading immune protective molecules of the host or leading to an immunomodulation of the host´s immune response [50].

PC has been recognized as a widespread antigenic determinant in many important disease-causing pathogens, comprising bacteria [51], nematodes [8] and also protozoa belonging to the genera *Trypanosoma* and *Leishmania* [9].

The PC modification of biomolecules in pathogens fulfills various functions for the organisms. For nematodes, it could be shown that the modification is important for development and fertility [20]. Bacteria use PC modifications for better adherence to epithelia by binding to the PAF receptor [52]. However, the most important function during infection is the modulation of the host´s immune system by PC modifications to escape the immune response.

PC-bearing antigens have been found to possess immunomodulatory capacity and to interfere with key proliferative signaling pathways in B- and T-cells, dendritic cell maturation and mast cell degranulation, thus facilitating the survival of parasites within their hosts [8,10,11,12,13,14,15,16,17].

Detailed data on the different types of PC-carrying compounds as well as their biosynthesis, however, are limited and have only been reported in the last few years [8,18,19,20,21].

So far, most PC epitopes investigated revealed glycan-bound PC-moieties. In multicellular parasites, PC has been found on *N*-glycans [21,35] and glycosphingolipids [53]. Additionally, in bacteria, PC was also found on lipopolysaccharide molecules e.g., in *Haemophilus influenzae* [54,55,56,57,58,59,60]. Furthermore, there is evidence for direct modification of amino acids as reported for the Rab1 protein at Ser^76^ by *Legionella pneumophila* [61]. However, there is indication that PC might be also bound directly to the protein backbone [62,63]. In eF1α from *L. infantum*, we found no evidence for glycosylation. This confirms our observations from its homologue in *A. suum*: here too, the PC seems to be directly coupled to the sidechains of amino acids [47].

Our proteomic study of *L. infantum* promastigotes revealed the presence of two spots containing eEF1α with similar pIs and molecular weights [64]. The ellipsoid form of the PC-positive spot in our study (see Figure 1B) and the two distinct spots in the Western blot using the anti-eEF1α antibody (see Figure 3A) confirmed these findings. Obviously, both protein species of eEF1α in these spots carry the PC modification (see Figure 3B). This raises the possibility that the protein might carry additional post-translational modifications. We and others [65] have found no indication for glycosylation; however, a tyrosine phosphorylation of eEF1α is reported for *L. donovani* [65]. 

The biosynthesis of the PC modifications still exhibits open questions since at least for non-bacterial pathogens neither the respective PC-transferase nor the PC-donor could be identified so far [66]. For *C. elegans*, some putative PC transferases have been postulated [67] by sequence homology to the PC transferase licD from *H. influenza* but they still await confirmation. For the same organism, a PC transferase activity was reported in microsomal preparations indicating phosphatidylcholine as a potential donor for the PC residues [18]. Since only eEF1α was found to be PC-positive, a quite specific transferase reaction might be postulated for *L. infantum*. Again, this transferase might be an interesting target for therapeutic intervention to treat *Leishmania* infections.

### 4.2. The Role of iEF1α in Parasitism

The eukaryotic elongation factor 1 alpha (eEF1α) is a housekeeping enzyme that catalyzes the GTP-dependent binding of aminoacyl-tRNA to the A-site of ribosomes during protein synthesis and is involved in the capture of deacylated tRNA [41,42]. The localization to the nucleus led to the suggestion that it may regulate transcription [68]. Furthermore, eEF1α was found to serve as a central hub in protein networks with hundreds of interacting partners [43,44]. eEF1α mRNA and protein levels are up-regulated in the stationary phase compared to the logarithmic phase of promastigotes [69].

eEF1α can be considered as a virulence factor in leishmaniasis. *Leishmania* eEF1α but not host derived eEF1α was found to bind and activate the Src-homology 2 domain containing protein tyrosine phosphatase-1 (SHP-1). SHP-1 is then involved in the pathogenesis of *Leishmania* infection through macrophage inactivation [41] resulting in the progression of leishmaniasis. Additionally, eEF1α blocks the induction of iNOS in response to interferon-γ.

The activation of SHP-1 by eEF1α resembles the up-regulation of SHP-1 by ES-62 in B-cells [70] both being dependent on the PC modification of the respective protein. On the intracellular level, ES-62 selectively targets multiple key signalling pathways following B-cell receptor ligation. By induction of the tyrosine phosphatase SHP-1, which dephosphorylates immuno-receptor tyrosine-based activator motifs (ITAMs), ES-62 prevents recruitment of the Ras/Erk MAP kinase cascade [70,71]. Moreover, recruitment of GAP and the dual phosphatase Pac-1 terminates coupling of B-cell receptor (BCR) signalling to Ras and Erk. ES-62 also negatively modulates the activation of the MAP kinase subfamilies, p38 and c-Jun N-terminal kinase (JNK) [72]. In addition, ES-62 selectively modulates the expression and activity of certain protein kinase C (PKC) isoforms [71,73].

eEF1α was found in early infection in *Leishmania* exosomes and identified as an important factor for immunosuppression and priming the host cells for *Leishmania* invasion [41,45]. These exosomes may suppress TNF-α production of infected monocytes but enhance production of the anti-inflammatory cytokine IL-10. Again, this resembles the biological PC-dependent activity of the immunomodulatory compound ES-62 from the nematode *A. viteae*.

In summary, these results provide new data for better understanding the biological function of eEF1a as a virulence factor in leishmaniasis.
